# Supplementary material for: Exploring the Associated Genetic Causes of Diabetic Retinopathy as a Model of Inflammation in Retinal Diseases
Source: Int J Mol Sci. 2024 May 17;25(10):5456. doi: 10.3390/ijms25105456 (PMC11121794; doi:10.3390/ijms25105456)
Supplement: Supplementary file 1 [file ijms-25-05456-s001.zip › Table S3.pdf]

Abbreviation	Full Description
TRAV23DV6	T cell receptor alpha variable 23/delta variable 6
TRAV3	T Cell Receptor Alpha Variable 3
IER3	Immediate Early Response 3
TRAV12-2	T Cell Receptor Alpha Variable 12-2
C4A	Complement C4A
TRBV18	T Cell Receptor Beta Variable 18
TRBV15	T Cell Receptor Beta Variable 15
FLOT1	Flotillin 1
HLA-DQA2	Major histocompatibility complex, class II, DQ alpha 2
TRAV17	T Cell Receptor Alpha Variable 17
HLA-DRB5	Major histocompatibility complex, class II, DR beta 5
C2	Complement C2
LINC00243	Long Intergenic Non-Protein Coding RNA 243
VARS2	valyl-tRNA synthetase 2, mitochondrial
SAPCD1-AS1	SAPCD1 antisense RNA 1
CD8A	CD8 subunit alpha
TRAV2	T Cell Receptor Alpha Variable 2
TRBV6-1	T Cell Receptor Beta Variable 6-1
TRAV12-1	T Cell Receptor Alpha Variable 12-1
ADGRG1	Adhesion G Protein-Coupled Receptor G1
TRIM10	Tripartite Motif Containing 10
TRBV10-3	T Cell Receptor Beta Variable 10-3
HSPA1B	Heat shock protein family A (Hsp70) member 1B
DCLRE1B	DNA Cross-Link Repair 1B
VARS1	Valyl-tRNA Synthetase 1
GPANK1	G-patch domain and ankyrin repeats 1
TRAV27	T Cell Receptor Alpha Variable 27
JUNB	JunB proto-oncogene
TRBV2	T Cell Receptor Beta Variable 2
ZFP1	ZFP1 zinc finger protein
TRAV8-1	T Cell Receptor Alpha Variable 8-1
TRAV20	T Cell Receptor Alpha Variable 20
RIOK3	RIO Kinase 3
AGER	Advanced Glycosylation End Product-Specific Receptor
BAG6	BAG cochaperone 6
HLA-DQB1-AS1	HLA-DQB1 antisense RNA 1
TNXB	Tenascin XB
TRBV6-5	T Cell Receptor Beta Variable 6-5
PARVA	Parvin Alpha
ACTN1	Actinin Alpha 1
FBXL22	F-Box And Leucine-Rich Repeat Protein 22
ZFYVE28	Zinc finger FYVE-type containing 28
TSBP1-AS1	Testis Specific Protein, Y-Linked 1 Binding Protein 1- Antisense RNA 1
TRBV5-6	T Cell Receptor Beta Variable 5-6
NFAT5	Nuclear Factor Of Activated T-Cells 5
CCHCR1	Coiled-Coil Alpha-Helical Rod Protein 1
PSORS1C3	Psoriasis Susceptibility 1 Candidate 3
